# Histopathological features of breast tumours in *BRCA1*, *BRCA2 *and mutation-negative breast cancer families

**DOI:** 10.1186/bcr953

**Published:** 2004-11-19

**Authors:** Hannaleena Eerola, Päivi Heikkilä, Anitta Tamminen, Kristiina Aittomäki, Carl Blomqvist, Heli Nevanlinna

**Affiliations:** 1 Department of Oncology, Helsinki University Central Hospital, Helsinki, Finland; 2 Department of Pathology, Helsinki University Central Hospital, Helsinki, Finland; 3 Department of Obstetrics and Gynaecology, Helsinki University Central Hospital, Helsinki, Finland; 4 Department of Clinical Genetics, Helsinki University Central Hospital, Helsinki, Finland

**Keywords:** breast cancer, oestrogen receptor, hereditary, p53, progesterone receptor

## Abstract

**Introduction:**

Histopathological features of *BRCA1 *and *BRCA2 *tumours have previously been characterised and compared with unselected breast tumours; however, familial non-*BRCA1/2 *tumours are less well known. The aim of this study was to characterise familial non-*BRCA1/2 *tumours and to evaluate routine immunohistochemical and pathological markers that could help us to further distinguish families carrying *BRCA1/2 *mutations from other breast cancer families.

**Methods:**

Breast cancer tissue specimens (*n *= 262) from 25 *BRCA1*, 20 *BRCA2 *and 74 non-*BRCA1/2 *families were studied on a tumour tissue microarray. Immunohistochemical staining of oestrogen receptor (ER), progesterone receptor (PgR) and p53 as well as the histology and grade of these three groups were compared with each other and with the respective information on 862 unselected control patients from the archives of the Pathology Department of Helsinki University Central Hospital. Immunohistochemical staining of erbB2 was also performed among familial cases.

**Results:**

*BRCA1*-associated cancers were diagnosed younger and were more ER-negative and PgR-negative, p53-positive and of higher grade than the other tumours. However, in multivariate analysis the independent factors compared with non-*BRCA1/2 *tumours were age, grade and PgR negativity. *BRCA2 *cases did not have such distinctive features compared with non-*BRCA1/2 *tumours or with unselected control tumours. Familial cases without *BRCA1/2 *mutations had tumours of lower grade than the other groups.

**Conclusions:**

*BRCA1 *families differed from mutation-negative families by age, grade and PgR status, whereas ER status was not an independent marker.

## Introduction

Women predisposed to hereditary or familial breast cancer form a heterogeneous group. It would be useful if we could identify carriers of the high-risk *BRCA1 *and *BRCA2 *genes and target the expensive and time-consuming genetic testing to individuals who most probably carry those mutations. Besides family history, histopathological markers could also be useful in distinguishing patients and families likely to carry a *BRCA1/2 *germline mutation from mutation-negative families and breast cancer patients in general.

Several studies have compared the characteristics of breast cancers in *BRCA1 *carriers and in sporadic controls. Distinct features between *BRCA1*-associated tumours have been found, such as high tumour grade, oestrogen receptor (ER) negativity, and overexpression of p53 [1-3]. In addition, negativity for progesterone receptor (PgR) [3,4], a higher proportion of medullary and atypical medullary carcinomas [5,6], and tumours with a low expression of c-erbB-2 [1,4,6] have been detected. Besides the higher proportion of medullary histology, a higher frequency of ductal carcinoma has also been reported [7,8]. Recently, cDNA expression analyses have suggested a basal epithelial phenotype for *BRCA1 *tumours [9] and expression of cytokeratins 5/6 has been associated with *BRCA1 *tumours [10].

Among *BRCA2*-associated tumours, a slight increase has been observed in the incidence of lobular or tubulolobular carcinomas [11,12]. However, results are inconsistent, and in most cases no significant difference has been found between *BRCA2*-associated tumours and sporadic cancers [1,4,6,13]. Complementary DNA (cDNA) expression analysis has suggested distinct expression profiles for *BRCA2 *tumours as well [14].

It has been clear for some time that in many families hereditary susceptibility is not due to the *BRCA1 *or *BRCA2 *genes [15,16]. Only a few studies have evaluated the features of this large group of families. It would be crucial for genetic counselling and for our understanding of tumour development to learn more about these patients. Lakhani and colleagues [17] studied the pathology of 82 familial breast cancers not attributable to *BRCA1 *or *BRCA2 *mutations, and they found these non-*BRCA1/2 *cancers to be of lower grade, to show less pleomorphism and to have a lower mitotic count than sporadic cancers or *BRCA *mutation-positive cancers. No other features differed significantly in their study. However, they did not examine immunohistochemical characteristics. There has been only one study so far that has characterised the immunohistochemical features of familial non-*BRCA1/2 *cancers: Palacios and colleagues [18] studied immunohistochemical staining and histopathology and compared 37 non-*BRCA1/2 *cancers with 20 *BRCA1*-associated and 18 *BRCA2*-associated cancers, and also with unselected control cancers. They similarly found those to be of lower grade than in all the other three groups. In comparison with sporadic cancers they were also more frequently p53-negative and erbB2-negative, and expressed reduced E-cadherin and β-catenin. However, the number of patients in this study was small and it was restricted to only a univariate analysis of the studied parameters.

Most of the previous studies on *BRCA1*-associated and *BRCA2*-associated cancers have studied highly selected patient groups, but in the present study families were collected with a simple criterion of at least three first-degree or second-degree relatives with breast or ovarian cancer with no restriction on age. We studied an extensive material of 152 non-*BRCA1/2 *tumours, and also 110 tumours from *BRCA1/2 *families for histopathological features as well as for the immunohistochemical expression of ER, PgR, p53 and erbB2. We describe here the histopathological profile of the tumours originating from non-*BRCA1/2 *breast cancer families and also present a multivariate analysis to find the independent markers that can further help in distinguishing especially *BRCA1 *mutation-positive families from other familial cases.

## Patients and methods

Familial breast cancer patients were identified and collected by a systematic screening for family history at the Department of Oncology, Helsinki University Central Hospital, as described previously [19]. We defined breast cancer families by the selection criterion of at least three first-degree or second-degree relatives with breast or ovarian cancer (including the proband). We confirmed the genealogy of the families through population registries, and cancer diagnoses through the Finnish Cancer Registry. In this study we included 25 *BRCA1 *families, 20 *BRCA2 *families and 74 families not associated with either of these genes (non-*BRCA1/2 *families) (Table 1). All families had previously been tested for *BRCA1 *and *BRCA2 *mutations by mutation analysis of the whole coding sequences and exon/intron boundaries of the genes as described [16,20] or tested for all previously reported 18 Finnish *BRCA1 *and *BRCA2 *mutations [16,20-22].

We collected all the paraffin blocks of all the primary breast cancers that were available (*n *= 262) from these families. However, cases tested to be non-carriers in the mutation-positive families were excluded. In total, 51 cancers from the 25 *BRCA1 *families, 59 cancers from the 20 *BRCA2 *families and 152 cancers from the 74 non-*BRCA1/2 *families were included in this study. We studied the haematoxylin and eosin sections of the original blocks to achieve histological diagnosis and grading. Grading was performed according to Scarff-Bloom-Richardson modified by Elston and Ellis [23]. The most representative area of the tumour was punched to produce a hereditary breast cancer tissue microarray including two cores (diameter 0.6 mm) from all of the original blocks. The array block of non-*BRCA1/2 *cases was described previously by Vahteristo and colleagues [24]. All of the microarray slides were stained with routine methods by antibodies against ER, PgR, p53 and erbB2 in the same pathology laboratory as our controls. In brief, 5 μm sections were cut from paraffin-embedded blocks, deparaffinated in xylene, and dehydrated in a series of graded alcohols. The sections were pretreated in a microwave oven and incubated overnight with antibody. Antibodies for ER (dilution 1:50) and c-erbB-2 (NCL-CB11; dilution 1:400) were purchased from Novocastra (Newcastle upon Tyne, UK), and those for PgR (dilution 1:250) and p53 (dilution 1:100) were from Dako (Copenhagen, Denmark). The evaluation of the staining results was similar to that used in routine diagnostics and samples were considered positive when 10%, 10% and 20% of the cells were stained for ER, PgR and p53, respectively. Samples having a moderate or intense staining of the entire membrane in more than 10% of the tumour cells (2+ and 3+) were considered to be c-erbB-2-positive. Other staining patterns were considered to be negative (0 and 1+).

As a control group we drew from the archives of the Pathology Department at Helsinki University 862 unselected breast cancer tumours from the years 1997–2001 that were scored by the same pathologist (PH) as the tumours of the hereditary breast cancer patients.

Statistical analysis was conducted with SPSS version 8.0 for Windows. We tested the differences in continuous variables by the Mann–Whitney test and in dichotomous variables by the χ^2 ^test or Fisher's exact test. In multivariate analysis, we used logistic regression analysis (stepwise backwards logistic regression, 99%). All *P *values are two-sided.

Permissions for this study were obtained from the ethics committees of the Department of Oncology and the Department of Obstetrics and Gynaecology, Helsinki University Central Hospital, and of the Ministry of Social Affairs and Health in Finland. Blood and tumour samples were used in this study with written informed consent from probands and family members.

## Results

*BRCA1*-associated cancers were diagnosed at a younger age than unselected breast cancers (median ages 44 and 56 years, respectively; *P *≤ 0.0005) and they were more often ER-negative and PgR-negative, p53-positive and of higher grade (Table 2). The frequency of medullary histology was also higher. In logistic regression analysis, taking into account all of these factors, the independent factors were age (*P *≤ 0.0005), ER status (*P *= 0.0597), PgR status (*P *= 0.0170) and medullary histology (*P *= 0.0636).

In comparison with familial non-*BRCA1/2 *cancers (median age 55 years), a univariate analysis of *BRCA1 *tumours showed similar differences from unselected cancers (Table 2). However, in multivariate analysis, taking into account the same factors, the independent factors were age (*P *= 0.0012), grade (*P *= 0.0014) and PgR negativity (*P *= 0.0196) (Table 3). We did not find any significant differences between groups of tumours from carriers with different mutations or when comparing tumours from the carriers of the mutations at the 5' end or the 3' end of the gene.

*BRCA2*-associated cancers were diagnosed at younger age (median age 47 years) than unselected breast cancers (median age 56 years, *P *≤ 0.0005). They were also more often ER-negative and PgR-negative (Table 2). In multivariate analysis, the independent factors were age (*P *≤ 0.0005), PgR status (*P *= 0.0365) and p53 status (*P *= 0.0318).

When compared with familial non-*BRCA1/2 *cancers, the *BRCA2*-associated cancers were diagnosed at a younger age (median age 47 years; among non-*BRCA1/2 *patients the median age was 55 years; *P *≤ 0.0005). No other variable differed significantly from non-*BRCA1/2 *cancers (Table 2). We did not find any significant differences between tumours originating from different mutation carriers or when comparing tumours from the carriers of mutations from the OCCR (Ovarian Cancer Cluster region) (*n *= 7) and the end of the gene. In a logistic regression analysis comparing *BRCA2*-associated cancers with non-*BRCA1/2 *cancers and taking into account age, grade and all tested histological and immunohistochemical factors, the only independently significant factor was age (*P *= 0.0001) (Table 2).

Among familial non-*BRCA1/2 *patients, the median age of onset was marginally younger (55.0 years) than among the unselected controls (56.0 years, *P *= 0.060). The frequency of grade I and II tumours was much higher among the non-*BRCA1/2 *group than in the unselected control group (odds ratio 1.8, *P *= 0.009) (Table 2) or in the *BRCA1 *and *BRCA2 *groups. In a multiple regression analysis comparing non-*BRCA1/2 *tumours with unselected controls, grade (odds ratio 0.54, *P *≤ 0.00005) and ER status (odds ratio 2.4 for negative ER status, *P *= 0.0006) were the independent significant factors.

The erbB2 results were very similar in the three groups of familial cases, with 18.6%, 15.1% and 17.4% of the *BRCA1*, *BRCA2 *and non-*BRCA1/2 *tumours, respectively, expressing the erbB2 antigen (Table 2).

## Discussion

Although genetic testing for *BRCA1 *and *BRCA2 *mutations is available, it is expensive, time-consuming and stressful to patients. Several models have therefore been developed for evaluating the probability of carrying a *BRCA1 *or *BRCA2 *mutation [25-30]. In our previous study of Finnish breast cancer families [15,29], multivariate analysis suggested simple family history criteria for breast cancer onset under the age of 40 years and the presence of ovarian cancer to be most strongly associated with *BRCA1/2 *mutation status [29]. However, in addition to family history, it is important to find other markers that could help to identify mutation carriers. In many countries, markers that are already routinely used, such as ER, PgR, p53 and the grade of the tumour, could serve as an excellent tool to aid distinguishing families because this information is easily available. In this study, we found that *BRCA1*-associated cancers have a different histological profile and can be distinguished from other familial cancers. Specifically, multivariate analysis revealed age, grade and PgR negativity as the independent factors distinguishing *BRCA1 *tumours from familial non-*BRCA1/2 *tumours. *BRCA2*-associated cancers did not differ greatly, but those were also significantly younger and there was a trend towards higher grade than among familial non-*BRCA1/2 *cancers.

ER negativity has previously been highlighted to be linked to *BRCA1 *tumours. It was obvious in this study too. Our results are consistent with, for example, the study of Lakhani and colleagues [4], in which *BRCA1 *cancers were clearly more ER-negative (90%) than unselected control cancers (35%). However, in our study the overall percentages of ER-negative cancers were much lower for both groups (67% among *BRCA1 *and 19% among unselected controls). This might be due to a different age distribution because the prevalence of ER-negative cases is higher among young breast cancer patients [31,32] and our familial cases were not selected on the basis of young age of diagnosis. Furthermore, there is a very high coverage mammography screening (in the general age group 50–60 years) in Finland, aiding in finding asymptomatic cancers, which are more often ER-positive as well as being of lower grade and PgR-positive [33].

In this study, we specifically sought to compare *BRCA1 *cancers with familial non-*BRCA1/2 *cancers as well, which is the relevant question in a clinical and genetic counselling setting. Surprisingly, in this analysis the ER status was not an independent marker in multiple regression. More important markers were age, PgR status and grade. ER status was dependent on age and grade. Palacios and colleagues [18] also compared *BRCA1 *cases with non-*BRCA1/2 *cases (cases from families with at least three affected with breast cancer, one of them diagnosed under 50 years of age). In a univariate analysis, the results showing ER and PgR negativity, p53 overexpression and high grade were very similar to ours. However, multivariate analysis was not used for evaluating independent markers. Recently, cDNA expression analyses have suggested a basal epithelial phenotype for *BRCA1 *tumours [9]. Epithelial phenotype is associated with breast cancers that express neither ER nor erbB2, a feature that also occurs in *BRCA1*-mutation carriers [10]. A large majority (20 of 22 tumours) of *BRCA1*-associated tumours were also ER-negative and erbB2-negative in our study, in accordance with the high frequency of ER-negative tumours among *BRCA1 *carriers. The frequency of erbB2 expression was similar in all three groups of familial tumours.

*BRCA2*-associated tumours did not differ significantly from familial non-*BRCA1/2 *tumours, although they were diagnosed at an earlier age. Our results on *BRCA2 *cancers were quite similar to those of Palacios and colleagues [18] and Lakhani and colleagues [17], although in the former study the *BRCA2 *cancers were more ER-positive and PgR-positive (on the basis of smaller sample set of 14 cases).

*BRCA1 *and *BRCA2 *mutations have been detected in quite a low proportion of breast cancer families. In Finland, among families with three affected first-degree or second-degree relatives without age restrictions, the proportions were 10% and 11% for *BRCA1 *and *BRCA2*, respectively [16,20]. Thus an important and large group of families is not due to mutations in the *BRCA1 *and *BRCA2 *genes. In this study, the familial non-*BRCA1/2 *cancers were diagnosed at a marginally younger age than those among unselected cases, and were more often of lower grade than the control cancers or *BRCA1 *and *BRCA2 *cancers. These factors might be influenced by recall bias because patients having affected relatives undergo diagnostic procedures earlier than women with no family history of breast cancer. However, no such influence is seen for *BRCA1 *or *BRCA2 *cases, which often have a much stronger family background of cancer. Furthermore, a lower grade among familial non-*BRCA1/2 *cases has been detected elsewhere as well [17,18].

In comparison with *BRCA1 *or *BRCA2 *cancers, familial non-*BRCA1/2 *cancers represented a much greater number of ER-positive and PgR-positive cases; however, in comparison with the unselected control group they were more receptor-negative. The frequency of ER-positive cancers among our control cancers is quite high, which might be due to a later year of diagnosis than for non-*BRCA1/2 *cancers and in general because of age distribution [34], mammography screening [33] or ethnic differences [35] in different populations.

## Conclusions

In this report we have characterised familial non-*BRCA1/2 *tumours and evaluated routine immunohistochemical and pathological markers that could help us to further distinguish families carrying *BRCA1/2 *mutations from other breast cancer families. It is noteworthy here that, although ER negativity has been considered a hallmark of *BRCA1 *tumours, logistic regression analysis indicated that this was not an independent marker but was dependent on the age of diagnosis and tumour grade. When considering the possibility of mutation testing in the context of genetic counselling, for instance, it would be important to consider the tumour characteristics specifically in comparison with those from other breast cancer families. It also seems crucial to consider the histopathological features with regard to the age of the patients. In this study, the independent markers that distinguished *BRCA1 *carrier tumours from familial non-*BRCA1/2 *tumours were earlier age of diagnosis, negative PgR status and higher grade. These immunohistochemical and pathological characteristics of the tumours, which are available in routine pathological diagnostics, should be of value in evaluating the possibility of mutation and in targeting mutation screening in such families, especially when considering the characteristics of several tumours in the family and combined with the family history of cancer.

## Abbreviations

cDNA = complementary DNA; ER = oestrogen receptor; PgR = progesterone receptor.

## Competing interests

The author(s) declare that they have no competing interests.

## Figures and Tables

**Table 1:** Mutation spectrum and numbers of families and patients by mutation

Gene	Mutation	Number of families	Number of patients
*BRCA1*	ex 10 782 del AA	1	1
	ex 11 1047 C→T	1	1
	ex 11 1731 C→T	1	2
	ex 11 1806 C→T	1	1
	ex 11 1924 del A	1	1
	ex 11 2592 ins A	1	2
	ex 11 2803 del AA	1	3
	ex 11 3604 del A	3	3
	ex 11 3744 del T	2	2
	ex 11 3904 C→A	1	1
	ex 11 4153 del A	1	1
	int 11 4216 (- 2) A→G	4	9
	ex 13 4446 C→T	2	14
	ex 14 4599 G>T	1	3
	ex 17 5145 del 11	1	4
	ex 20 5370 C→T	1	1
	ex 20 5382 ins C	2	2
		
	Any	25	51
	
*BRCA2*	ex 9 999 del TCAAA	5	22
	ex 10 1822 G→T	1	1
	ex 11 4075 del GT	1	1
	ex 11 4081 ins A	1	2
	ex 11 5797 G→T	1	1
	ex 11 6496 del CA	1	2
	ex 11 6503 del TT	1	1
	ex 15 7708 C→T	3	12
	ex 18 8555 T→G	1	6
	int 23 9346 (- 2) A→G	5	11
		
	Any	20	59
	
Non-*BRCA1/2*		74	152

**Table 2:** Analysis of features among *BRCA1*-, *BRCA2*- and non-*BRCA1/2*-associated cancers and in unselected cancers

	*N *(frequency, %)				*P*
Feature	*BRCA1*	*BRCA2*	Non-*BRCA1/2*	Unselected	
Histology					
Ca ductale	37 (72.5)	37 (62.7)	102 (67.1)	561 (65.1)	
Ca lobulare	8 (15.7)	17 (28.8)	30 (19.7)	219 (25.4)	
Ca medullare	5 (9.8)	-	3 (2.0)	20 (2.3)	0.013*, 0.001**
Others	1 (2.0)	5 (8.5)	17 (11.2)	62 (7.2)	
	
Grade					
I	3 (6.1)	12 (23.1)	46 (32.4)	152 (23.3)	
II	11 (22.4)	26 (50.1)	66 (46.5)	289 (44.4)	
III	35 (71.4)	14 (26.9)	30 (21.1)	210 (32.3)	
I–II	14 (28.6)	38 (73.1)	112 (78.9)	441 (67.7)	≤ 0.0005*, <0.0005**, 0.009****
	
Immunohistochemistry					
ER^-^	28 (66.7)	17 (32.1)	40 (27.0)	167 (19.4)	≤ 0.0005*, <0.0005**, 0.026***, 0.035****
ER^+^	14 (33.3)	36 (67.9)	108 (73.0)	692 (80.6)	
PgR^-^	37 (84.1)	28 (51.9)	67 (45.3)	312 (36.2)	≤ 0.0005*, <0.0005**, 0.021***, 0.036****
PgR^+^	7 (15.9)	26 (48.1)	81 (54.7)	549 (63.8)	
p53^-^	27 (62.8)	42 (82.4)	118 (78.7)	628 (74.0)	0.034*, <0.0005**
p53^+^	16 (37.2)	9 (17.6)	32 (21.3)	221 (26.0)	
erbB2^-^	35 (81,4)	45 (84,9)	109 (82,6)	NA	
erbB2^+^	8 (18,6)	8 (15,1)	23 (17,4)	NA	

*BRCA1 versus non-*BRCA1/2 *tumours; **BRCA1 versus unselected breast tumours; ***BRCA2 versus unselected breast tumours; ****non-*BRCA1/2 *tumours versus unselected breast tumours.

Ca, carcinoma; ER, oestrogen receptor; NA, not available; PgR, progesterone receptor.

**Table 3:** Logistic regression analysis of features of breast cancers among *BRCA1*- and *BRCA2*-associated cases in comparison with non-*BRCA1/2 *cases

	Odds ratio (95% confidence interval, *P*)	
Feature	*BRCA1*	*BRCA2*
First step:		
Age (continuous)	0.94 (0.91–0.98, 0.0009)	0.94 (0.91–0.97, 0.0001)
Grade (continuous)	3.78 (1.70–8.39, 0.0011)	1.27 (0.71–2.26, 0.42)
ER^+^	1.0 (referent)	1.0 (referent)
ER^-^	0.91 (0.28–2.90, 0.88)	0.85 (0.32–2.29, 0.75)
PgR^+^	1.0 (referent)	1.0 (referent)
PgR^-^	4.18 (1.26–13.87, 0.019)	1.82 (0.75–4.38, 0.18)
p53^+^	1.0 (referent)	1.0 (referent)
p53^-^	1.96 (0.74–5.18, 0.18)	1.61 (0.63–4.12, 0.34)
	
Final step after stepwise regression:		
Age (continuous)	0.94 (0.92–0.98, 0.0012)	0.94 (0.92–0.97, 0.0001)
Grade (continuous)	3.25 (1.58–6.69, 0.0014)	
PgR^+^	1.0 (referent)	
PgR^-^	3.45 (1.22–9.80, 0.02)	

ER, oestrogen receptor; PgR, progesterone receptor.
